# Well-being interventions in U.S. colleges: a scoping review from a positive higher education perspective

**DOI:** 10.3389/fpsyg.2025.1601955

**Published:** 2025-08-29

**Authors:** Grace X. Zhao, Maggie Y. Zhao, Caitlin R. Bean, Ashley S. Robbins, Nicole E. Mahrer

**Affiliations:** ^1^General Education Program, Music Department, University of La Verne, La Verne, CA, United States; ^2^Teaching and Learning Innovation Centre, The University of Hong Kong, Pokfulam, Hong Kong SAR, China; ^3^Psychology Department, University of La Verne, La Verne, CA, United States

**Keywords:** well-being, higher education, positive education, positive higher education, well-being interventions

## Abstract

In response to growing concerns about student mental health, this scoping review synthesizes 40 peer-reviewed empirical studies published between 2009 and 2024 on well-being interventions for undergraduate students in U.S. higher education, examined through the emerging lens of Positive Higher Education. Drawing from diverse methodologies and interventions—including mindfulness, cognitive-behavioral therapy, psychoeducation, and positive psychology strategies—the review examines how well-being is defined, implemented, and assessed across curricular and co-curricular contexts. While many interventions demonstrated positive outcomes, particularly in reducing stress and enhancing psychological well-being, the field remains limited by conceptual ambiguity, inconsistent theoretical frameworks, and insufficient attention to long-term institutionalization and sustainability. Most studies lacked a clearly articulated definition of well-being, and relatively few were embedded in academic curricula. This review underscores the need for a coordinated, theory-informed approach to integrating well-being as a core educational outcome, aligned with the values of liberal arts education. It concludes with a call to develop a Positive Higher Education Implementation Toolkit to guide institutions in embedding well-being into policy, pedagogy, and practice.

## 1 Introduction

### 1.1 Student mental health crisis

The phrase “mental health crisis among college students in America” has become a prominent feature in both scholarly literature and public discourse. While a Google Scholar search using this exact phrase yields over two million results—an unscientific method, to be sure—it nonetheless reflects the magnitude of concern surrounding this issue. Empirical data from the American College Health Association’s National College Health Assessment (American College Health Association, 2021, 2022, 2023, 2024, 2025) surveys conducted between Fall 2020 and Fall 2024 substantiate the crisis narrative, revealing consistent and troubling patterns in college student mental health. Rates of serious psychological distress remained relatively stable, though elevated, with a modest decline observed in Spring 2024 (19.5%) compared to Spring 2021 (24.0%). Loneliness affected approximately half of all students each term, peaking at 53.0% in Spring 2021 before slightly declining to 48.5% in Spring 2024. Diagnoses of anxiety and depression showed a more pronounced upward trend, with anxiety increasing from 29.6% in Fall 2020 to 35.0% in Fall 2024, depression from 22.0 to 25.3%, and comorbidity of both conditions rising from 18.3 to 24.3%.

While the COVID-19 pandemic is often cited as a catalyst for worsening mental health outcomes, research indicates that many of these challenges preceded the pandemic and have been driven by systemic stressors such as academic demands, financial insecurity, and social pressures (Kruisselbrink Flatt, 2013; Lipson et al., 2019). A particularly concerning finding from the ACHA-NCHA III data is that nearly half of all students reported experiencing at least 3 days in the past month when mental health concerns interfered with their academic performance (American College Health Association, 2024, 2025). These findings underscore not only the persistence of the student mental health crisis but also its direct impact on academic success, retention, and the broader mission of higher education.

### 1.2 Literature review of recent reviews of well-being interventions for college students

Institutions of higher education—and their faculty, staff, and administrators—have become increasingly attuned to the escalating mental health crisis among college students. In response, a diverse range of well-being interventions has been implemented, paralleled by a growing body of empirical research aimed at evaluating their effectiveness. A bibliometric mapping study by Hernández-Torrano et al. (2020), which analyzed 5,561 articles across psychology, psychiatry, and education disciplines (predominantly from the United States), documented a sharp increase in scholarly attention to student mental health and well-being, underscoring higher education’s emergence as a critical environment for advancing student well-being.

Recent systematic reviews affirm the efficacy of a variety of interventions in this domain (Barnett et al., 2021; Conley et al., 2015, 2016, 2017; Fenton et al., 2018; Winzer et al., 2017, 2018). Particularly, systematic reviews of reviews conducted by Worsley et al. (2020, 2022) identified mindfulness-based interventions (MBIs), cognitive-behavioral therapy (CBT), psychoeducational programs, and stress management techniques as widely used approaches, with MBIs and CBT demonstrating particularly strong outcomes in reducing anxiety and depression. Complementing these reviews, du Toit et al. (2022) conducted a meta-analysis of 62 longitudinal studies and identified personal (e.g., mindfulness, self-regulation), relational (e.g., social support, reduced loneliness), and contextual (e.g., financial stability) factors as significant predictors of student well-being. These findings underscore the multidimensional nature of well-being and the need for comprehensive, multilevel intervention strategies.

Research across global higher education contexts reveals both contextually grounded insights and recurring challenges in the design and implementation of student well-being interventions. In a scoping review of Australian universities, Gilmore et al. (2025) identified a wide range of psychological, social, institutional, and lifestyle factors shaping student well-being, while also noting methodological limitations and a lack of campus-specific initiatives. Similarly, Hall et al. (2024) examined the application of positive psychology frameworks in Asian higher education, highlighting their potential to foster leadership, engagement, and academic achievement. However, their review underscored the limited quality of empirical evidence and the insufficient integration of personal strengths into educational practice. In the United Kingdom, Dodd et al. (2021) found considerable inconsistencies in how well-being is conceptualized and measured across studies, including a lack of coherent theoretical frameworks and the use of diverse assessment tools, few of which were developed or validated for the particular student populations.

Additionally, an important perspective emerging from the review literature is offered by Hernández-Torrano et al. (2020), who argue that much of the existing scholarship remains anchored in a pathogenic paradigm—one that prioritizes the diagnosis and treatment of mental illness. They propose a shift toward holistic, salutogenic approaches that conceptualize well-being as dynamic, multidimensional, and oriented toward the promotion of health and flourishing.

### 1.3 Conceptualizing student well-being through the lens of positive psychology

Positive psychology offers a perspective that challenges conventional deficit-based approaches, which primarily focus on identifying and addressing mental illness. It emphasizes a strengths-based approach that builds happiness, cultivates strengths, and promotes holistic flourishing (Seligman and Csikszentmihalyi, 2020). This view is supported by empirical evidence; for instance, Zhao and Tay (2023) demonstrate that well-being and ill-being exist along a continuum with each representing its own distinct and unique domain. Building on this foundation, the Positive Education framework provides a structured, evidence-based approach for integrating well-being with educational outcomes. Introduced by Seligman et al. (2009), Positive Education positions student well-being as essential to academic success by aligning traditional educational goals with the principles of positive psychology. In K–12 settings, this model is often enacted through a whole-school approach that incorporates strengths-based learning, character development, and social–emotional learning into curricula, policies, and institutional culture (Seligman and Adler, 2018). The International Positive Education Network (IPEN) further articulates this integration through the “Double Helix” framework, which emphasizes the reciprocal relationship between well-being and academic achievement (White and Kern, 2018).

While widely adopted in primary and secondary education, adapting Positive Education to higher education—potentially referred as “Positive Higher Education”—presents structural and cultural challenges. The decentralized governance of universities, disciplinary silos, and strong norms of faculty autonomy hinder the implementation of comprehensive, institution-wide strategies (Oades et al., 2011; Sweeting et al., 2023). Unlike K–12 systems, higher education institutions face competing priorities and fragmented organizational cultures that complicate efforts to embed well-being into the academic mission (Sweeting et al., 2023).

Nonetheless, growing concern over the mental health crisis in university populations has catalyzed increased attention to student well-being in higher education. The Okanagan Charter (2015) marked a pivotal development by calling on universities to become health-promoting institutions. It advocates for the integration of health and well-being across governance, operations, and academic priorities, reframing well-being as both a means and an end of higher education—essential for learning, civic engagement, and lifelong flourishing. This vision should particularly resonate with the aims of liberal education in the U.S., which prioritize holistic student development in preparation for a complex and interconnected world (Finley, 2016).

Despite increasing interest, Positive Higher Education remains an emerging paradigm lacking a clear and unified framework. Oades et al. (2011) argue that fostering a genuinely positive university requires integrating Positive Education principles with organizational well-being practices, echoing the holistic vision of the Okanagan Charter (2015). Rather than prescribing specific interventions, Positive Higher Education may be best understood as a perspective—an aspirational vision of higher education where student well-being is embedded into institutional values, structures, and practices. It reimagines universities as ecosystems for flourishing, in which intellectual, emotional, social, and ethical development are central to the educational experience and outcome.

While grounded in the foundational ideas of Positive Education (Seligman et al., 2009), Positive Higher Education differs from K–12 applications in both scope and implementation. K–12 settings typically adopt structured, teacher-led programs, whereas higher education emphasizes student autonomy and developmental appropriateness for emerging adults. Integration occurs through academic curricula, co-curricular programming, and institutional strategies such as policy frameworks, staff training, and cross-campus coordination. These multilayered approaches reflect a broader shift toward embedding well-being into the fabric of university life.

This perspective aligns with recent arguments by Kristjánsson and VanderWeele (2024), who elaborate that flourishing should be seen as the central aim of all educational endeavors. By integrating well-being across all facets of academic life, this paradigm positions flourishing as a fundamental outcome of higher learning.

### 1.4 The goals of this scoping review

This scoping review is inspired by Dodd et al.’s (2021) scoping review of student well-being in UK higher education by applying a similar approach to the U.S. context. The American undergraduate experience, grounded in liberal arts education, offers a distinctive lens through which to examine how student well-being is conceptualized, implemented, and strengthened. According to Merriam-Webster (n.d., “liberal education”), liberal education is “education based on the liberal arts and intended to bring about the improvement, discipline, or free development of the mind or spirit.” Exploring how well-being fits into this educational philosophy offers valuable insight into the broader mission of higher education.

A central aim of this review is to examine well-being interventions in U.S. colleges through the lens of *positive higher education,* an extension of the Positive Education paradigm first introduced by Seligman et al. (2009). Positive education emphasizes well-being as a core educational outcome—on par with academic achievement—and advocates for its integration through whole-institution approaches (White and Kern, 2018). While existing reviews often focus on methodological rigor or institutional case studies, there remains a need for a comprehensive synthesis of peer-reviewed research that examines well-being interventions within a unified, conceptual framework. This review seeks to address this gap by synthesizing empirical research on how well-being interventions are defined, implemented, and evaluated in U.S. higher education—particularly in undergraduate settings.

While faculty and staff well-being are essential to a healthy campus ecosystem, this review focuses specifically on undergraduate students, who face growing mental health challenges. This focus also aligns with a broader interest in understanding how well-being and mental health initiatives are embedded within the liberal education model prevalent in U.S. colleges and universities.

Guided by the emerging concept of Positive Higher Education, this scoping review seeks to provide researchers and practitioners with evidence-based, actionable insights into student well-being interventions within American higher education contexts. Building on the work of Dodd et al. (2021)—whose scoping review aimed to identify how well-being is defined, which indicators are used to measure it, and what instruments are employed among university students in the UK—this review adopts a similar aim but extends the focus to the American undergraduate context. Specifically, it emphasizes how well-being interventions are embedded within broader institutional features of U.S. higher education, including general education, liberal arts curricula, and shared student experiences.

Accordingly, this review examines:

- How well-being is defined and operationalized;
- What constitutes a well-being or mental health intervention;
- How interventions are integrated into the undergraduate experience;
- How outcomes are assessed and whether they align with intervention goals; and
- What strategies support the institutionalization and sustainability of well-being initiatives, particularly regarding institutional frameworks and funding.

A secondary aim is to leverage synthesized findings from these empirical studies to contribute to a deeper understanding of student well-being and to help shape the emerging vision of *positive higher education*.

## 2 Methods

The PICOS framework guided the development of the review protocol, including the identification and selection of studies and the determination of relevant data for extraction. A comprehensive search was conducted across major academic databases for peer-reviewed articles published in English between 2009 and Fall 2024. The starting point of 2009 was selected to align with the publication of Seligman et al.’s (2009) seminal paper on positive education, marking a foundational moment in the field.

To capture the diverse range of approaches to student well-being, this review included studies focused on mental health interventions, positive education-based programs, and targeted strategies such as character strengths interventions. Consistent with the review’s objectives, a singular definition of well-being was not imposed. Instead, the review was guided by an inclusive understanding of well-being as a multidimensional construct encompassing emotional, psychological, social, and physical dimensions of human functioning (e.g., Dodge et al., 2012; Ryff, 1989). This broad conceptual framing enabled an examination of how varying definitions of well-being inform the design, implementation, and evaluation of interventions within U.S. higher education settings. The review focused specifically on undergraduate students enrolled in U.S. colleges and universities (i.e., post-secondary students not enrolled in graduate or professional programs) to explore how well-being interventions are conceptualized and operationalized within the context of American liberal arts education. To maintain a clear and consistent scope, studies involving mixed student populations (e.g., both undergraduate and graduate students) were excluded. Full inclusion and exclusion criteria are presented in Table 1.

**Table 1:** Inclusion and exclusion criteria.

Criteria	Include	Exclude
Population	Post-secondary students enrolled in U.S. colleges or universities. Focus on undergraduate student populations (e.g., associate’s or bachelor’s degree programs).	Students at other education levels (e.g., primary or secondary) or other populations (staff, faculty, non-U.S.), students in graduate programs or professional programs
Intervention	Interventions aimed at cultivating well-being	Interventions targeting specific pre-existing mental health or neurodevelopmental conditions (e.g., ADHD, autism)
Comparison	All control or comparator groups, or studies without control groups	n/a
Outcomes	All mental health and well-being outcomes, including educational performance outcomes	n/a
Study Design	Peer-reviewed empirical research (including both quantitative and mixed methods)	Qualitative only methods, meta-analyses, systematic review, and others
Publication	English-language publications between 2009–2024	Studies published in other languages or outside the specified date range

Quantitative studies with and without control or comparison groups were included. Studies that used solely qualitative measures were excluded due to this review’s goal of examining methods of standardized measurement of well-being outcomes.

### 2.1 Search strategy

**Figure 1:**
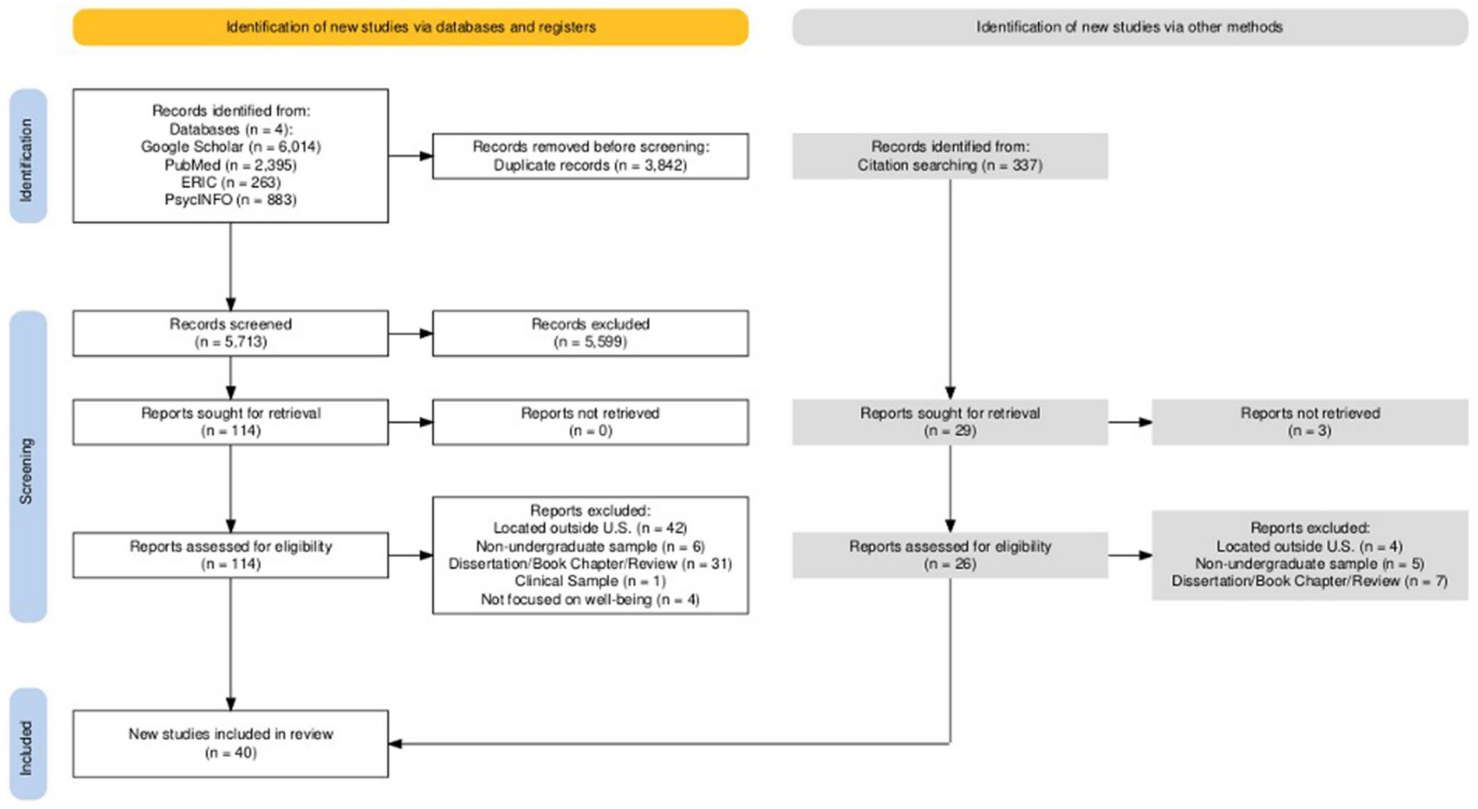
PRISMA 2020 flow diagram for new scoping reviews which included searches of databases and other sources (Page et al., 2021). Diagram generated by Haddaway et al. (2022).

Databases utilized in the search included Google Scholar, PubMed, ERIC, and PsycINFO. A combination of several search terms were used via the advanced search options and Boolean operators. Specific search terms were: (1) Well-being interventions OR Mental-health interventions OR Well-being strategies OR Well-being campaigns OR Positive Psychology interventions OR Character Strength; (2) College OR University OR Higher Education OR Student; (3) US OR America. Search results appeared based on how closely the article matched the search terms and timeframe. The software Publish or Perish was used to download the first 1,000 relevant studies that appeared in the search results for each database then uploaded to Endnote for further screening. The total number of articles that were identified from each database can be seen in Figure 1. Any duplicates within databases and between databases were eliminated before screening in Endnote.

### 2.2 Study screening and selection

To identify eligible studies from the original search, two reviewers first screened and eliminated any studies with exclusion criteria present in the titles and abstracts. If abstracts met inclusion criteria, papers were sought for retrieval and read and analyzed for eligibility. At this stage, reviewers referenced the exclusion criteria to determine whether to include a study. Studies eligible for the review were included in the study for data extraction (see Figure 1). Other extraction methods utilized by the reviewers included searching through citations in reference sections of included articles. Similar to the database procedure, reviewers screened titles and abstracts of the referenced articles to determine if the article met eligibility. If the article passed the first screening, the reviewers sought the full text for retrieval to read. Reports were excluded based on inclusion and exclusion criteria in Table 1. In total, 30 articles were determined eligible for data extraction from databases, and 10 articles were eligible from citation searching.

### 2.3 Data extraction

Data were extracted independently by three reviewers using a standardized and piloted data extraction form, in accordance with PRISMA guidelines (2021). For each included study, the following information was recorded: publication details (authors and year), sample size, study duration, intervention type, outcome measures, and educational context (curricular/co-curricular). Interventions were additionally coded for delivery method (e.g., in-person, online, hybrid), theory of well-being/focus of the study, whether the intervention was part of an Institutional initiative, study design [randomized control trial (RCT), pre-post, or quasi experimental], and positive intervention effects.

A secondary data extraction phase was conducted to capture contextual and theoretical dimensions. Extracted variables included the presence and specification of a well-being theory or definition, the stated focus of the intervention (e.g., emotional, social, academic), the educational context (e.g., curricular, co-curricular), institutional context and support mechanisms (e.g., administrative leadership, faculty involvement), and whether the study reported external funding.

Discrepancies in data extraction were resolved through discussion among the reviewers, with consensus reached in all cases.

## 3 Results

A total of 40 empirical studies met the inclusion criteria, all conducted in the United States and focused exclusively on undergraduate student populations (details see Table 2). While the studies employed a variety of research designs—including randomized controlled trials, quasi-experimental methods, and pre–post intervention models—they all aimed to assess the effectiveness of one or more well-being or mental health interventions. Table 2 provides an overview of each study, detailing intervention types, sample size, study designs, outcome measures, outcomes, settings of integration into the undergraduate context, use of well-being-related theories or researcher-defined constructs, and whether the intervention was part of a broader institutional well-being framework or initiative. Summaries of the findings are organized below according to the five guiding research questions.

**Table 2:** Summary of interventions and outcomes in well-being studies.

Authors	Sample size	Intervention	Duration	Outcome measure	Educational context	Theory of well-being/focus	Institutional initiative	Study design	Positive outcomes
Baghurst and Kelley (2024)	531	Stress Management, Sport Activity, Fitness	16-week	PSS, TAS, PBS	Curricular (workshop)	NS/stress reduction	NS	Quasi-experimental	Decreased perceived stress, test anxiety and personal burnout in stress-management and physical activity groups; Decreased perceived stress and personal burnout in fitness group
Barker et al. (2016)	78	Therapy Dogs	15 min	PSS-10, SVAS, sNFG, sAA	Co-Curricular (interaction session)	NS/stress reduction	NS	RCT	Decreased stress in intervention group
Bergen-Cico et al. (2013)	119	Mindfulness-Based Stress Reduction	5-week	KIMS, PHLM, SCS, STAI-T	Curricular (workshop)	NS/mindfulness, awareness, self-compassion	NS	Quasi-experimental	Increased mindfulness, self-compassion in MBSR group
Browning et al. (2023)*	34	Acceptance & Commitment Therapy	5-week	DASS-21, Q-LES-Q-SF, UFM	Curricular (seminar)	NS/psychological flexibility, quality of life	NS	RCT	Increased well-being and mindfulness, decreased distress in intervention group
Burns et al. (2011)*	43	Meditation	2-semester	PSS-14, BAIT, CES-D, PCI	Co-curricular (workshop)	NS/stress reduction	NS	Pre-post	Decreased stress, trait anxiety, and perfectionist thinking
Caldwell et al. (2010)	166	Movement Classes, Physical Activity	15-week	FFMQ, PSQI, SRSE, FDMS, PSS-4	Curricular (workshop)	NS/awareness, stress reduction	NS	Quasi-experimental	Increased mindfulness, sleep quality in all groups
Caldwell et al. (2011)	76	Taijiquan	15-week	FFMQ, PSQI, SRSE, FDMS, PSS-4	Curricular (workshop)	NS/awareness, stress reduction	NS (Option to fulfill a GE Physical Education/Wellness Requirement)	Quasi-experimental	Increased mindfulness, improved sleep quality, mood, and decreased perceived stress in Taijiquan group
Conley et al. (2013)	51	Psychosocial Wellness	Academic year/8 months	SES-GSE, RSE, CD-RS, ADHS, SWLS, MAAS, ERQ (Reappraisal & Suppression subscale), ATQ-P, DASS-21, PSS, DAS, SACQ, ICSRLE	Curricular (workshop)	NS/psychological adjustment, stress management	NS	Quasi-experimental	Improved psychological adjustment and stress management in intervention group
Conley et al. (2024)	145	Online Mindfulness App	2-month	PHQ-9, RRQ, PSWQ, PSS-4, SHS, SCS-SF, CLS, FFMQ, SBI, SRSE	Co-curricular (phone application)	NS/distress reduction, positive well-being	NS	RCT	Decreased depression symptoms, anxiety, stress, and improved affect, positive well-being outcomes in intervention group
Costello et al. (2022)	438	Social Connection	Semester/16 weeks	BDI, UCLA Loneliness Scale, PSSM	Co-curricular (workshop)	Belongingness Theory/social connection	The Connection Project*	RCT	Increased school belonging, and decreased loneliness, depressive symptoms in intervention group
Dalton and Estrada (2023)	48	Mindfulness-based	4 weeks	FFMQ, CEI-II, PSS	Curricular (workshop)	NS/stress reduction, intellectual curiosity	NS	RCT	Increased trait mindfulness, weekly mindfulness practice, and lower perceived stress in intervention group
Dvorakova et al. (2017)*	109	Mindfulness	6 weeks	PHQ, GAD, SWLS, MAAS, SCS, SCC-R, CS, PSQI, YAAPST	Co-curricular (workshop)	NS/stress reduction, awareness	NS	RCT	Improved life satisfaction, and decreased depression, anxiety, sleep issues, and alcohol consequences in intervention group
Eustis et al. (2018)*	156	Acceptance-Based Behavioral Therapy	3×15 minutes over 4 weeks	DASS-21, BFNE, AAQ, PHLM, QOLI, VLQ, EQ	Co-curricular (workshop)	NS/anxiety, depression symptom reduction	NS	RCT	Decreased general anxiety in intervention condition
Feldman and Dreher (2011)	96	Hope Intervention/Goal Setting	90 min	GSHS, PILT, VIQ	Curricular (workshop)	Hope Theory/purpose, hope increase	NS	RCT	Increased hope, life purpose, progress towards a goal, and vocational calling in intervention group
Firestone et al. (2019)	163	Acceptance & Commitment Therapy	60–90 min	PVQ, PWB, ZKPQ-Form III Inf	Co-curricular (online workshop)	NS/value engagement, psychological adjustment	NS	Pre-post	Improved valued living
Flinchbaugh et al. (2012)	117	Stress Management, Gratitude Journaling	12-week	PSS, SWLS, +	Curricular (workshop)	NS/stress reduction, life satisfaction, engagement	NS	Quasi-experimental	Improved meaningfulness and engagement in combined intervention and gratitude journaling treatment conditions
Frazier et al. (2015)	257	Perceived Control on Stress	60 min	PSS, DASS-21, PCOSES	Curricular (online workshop)	The Temporal Model of Control/stress perception	NS	RCT	Improved present control, and decreased perceived stress, depression and anxiety in intervention group
Goodmon et al. (2016)*	38	Positive Psychology	A semester/16 weeks	AHI, GHQ, SWLS, AHQ, CES-D, PSS	Curricular (seminar)	NS/happiness, stress reduction	NS	Quasi-experimental	Increased happiness, life satisfaction, routes to happiness, and decreased depressive symptoms and stress in positive psychology group
Hendrickse et al. (2024)	216	Wellness, Resilience	4-week	Self-efficacy	Co-curricular (website)	NS/self-efficacy, resilience	Campus wide project (The student resilience project)	RCT	Increased self-help behaviors in high-exposure group, self-efficacy for students of color
Hintz et al. (2015)	292	Undergraduate Stress Psychoeducation		DASS-21, PSS, PCOSES	Co-curricular (online seminar)	The Temporal Model of Control/stress management	NS	RCT	Decreased stress, depression, and anxiety in intervention group
Huberty et al. (2019)*	88	Mindfulness	8 weeks	PSS, FFMQ, SCS-SF, PROMIS, YRBS	Co-curricular (phone application)	NS/stress reduction	NS	RCT	Decreased perceived stress, and increased mindfulness, and self-compassion in Mindfulness group
Jeong et al. (2022)*	42	Positive Psychology Robot	7 sessions	Mini-IPIP, RPWS, BMIS, RtCR, WAI-SR	Co-curricular (electronic workshop)	NS/overall positive psychology & well-being	NS	Quasi-experimental	Increased psychological well-being, mood, readiness to change in intervention group
Ko et al. (2018)*	41? (AC)	Compassion	15-week	FFMQ, SCS, CS, CES-D, STAI, PSS	Co-curricular/Curricular (seminar)	NS/mindfulness, compassion, self-compassion	NS	RCT	Increased mindfulness, self compassion, compassion, improved and bio-marker for stress in intervention group
Levin et al. (2016)*	234	Acceptance & Commitment Therapy—College Life	21 days	DASS-21, MHC-SF, AFQ-Y, PVQ, FFMQ	Co-curricular (online seminar)	NS/psychological flexibility	NS	Pre-post	Mental health outcomes largely equivalent to previously used intervention
Levin et al. (2017)*	79	Acceptance & Commitment Therapy	4 weeks	MHC-SF, AAQ-II, CFQ, VQ, PHLMS, CCAPS-34	Co-curricular (online seminar)	NS/psychological flexibility	NS	RCT	Decreased overall distress, anxiety, depression, academic concerns and improved positive mental health in intervention condition
Long et al. (2021)*	208	Mindfulness	6 weeks	MAAS, GLES, DERS-18, ATQ-SF, COPE, SCS-SF, PWB—Positive Relations with Others subscale, BRS	Co-curricular (workshop)	NS/emotion regulation, stress management	NS	Quasi-experimental	Improved mindfulness, executive control, coping, self-compassion, connectedness, resilience and flourishing in Mindfulness workshop
Maybury (2012)	23	Positive Psychology	12 weeks	MAAS, DES, MLQ, SWLS, AHS, SISA, SHS	Curricular (seminar)	NS/overall positive psychology & well-being	NS	Quasi-experimental	Increased hope, self-actualization, well-being, agency and purpose in intervention group
Melnyk et al. (2015)	121	Cognitive Behavioral Skill-Building	Semester	PBS, PHQ-9, GAD-7	Curricular (online workshop)	NS/depression, anxiety symptom reduction	NS	Quasi-experimental	Improved symptoms for participants with high baseline anxiety in CBT workshop
Nguyen-feng et al. (2017)	365	Stress Management	2-week	TLEQ, DASS-21, PSS	Co-curricular (online workshop)	NS/stress management	NS	Quasi-experimental	Decreased stress, anxiety, depression, and perceived stress in all intervention groups
Prestin and Nabi (2020)	295	Positive Media	5 days	AHS, +	Co-curricular (online videos)	Broaden-and-Build Theory, Mood Management Theory/positive emotion, stress reduction	NS	Quasi-experimental	Decreased stress and illness symptoms in intervention group
Ramler et al. (2015)*	62	Mindfulness-Based Stress Reduction	8-week	SACQ, FFMQ +	Curricular (workshop)	NS/stress reduction	NS	Quasi-experimental	Improved first-year student adjustment and reduced psychological stress in intervention group
Renshaw and Rock (2018)	97	Gratitude Thinking		GQ-6, SWLS, SHS, PANAS, DASS-21	Co-curricular (self-practice)	NS/gratitude	NS	RCT	Increased happiness, satisfaction, and decreased depression, stress, and negative affect in intervention group
Renshaw and Hindman (2017)	115	Gratitude Activities	2-week	UCLA Loneliness-R, DASS-21, SWLS, PSSM, LOT-R, GQ-6	Co-curricular (self-practice)	NS/gratitude	NS	RCT	Increased optimism and school connectedness in intervention group
Rust et al. (2009)	131	Character Strength	12-week	SWLS, VIA-IS	Curricular (workshop)	NS/strengths, weaknesses, life satisfaction	NS	Quasi-experimental	Improved life satisfaction in Character Strength workshop
Seppälä et al. (2020)*	790	Happiness, Mindfulness, or Emotional Intelligence	8-week	PSS-10, MASQ-D30, RAND-SF36, RPWS, SWLS, PANAS, GQ-6, SCS-SF, FFMQ-15, COPE, LOT-R, SISE, SCS-R, +	Co-curricular (workshop or seminar)	NS/resilience, thriving	SKY Campus Happiness, Foundations in Emotional Intelligence, Kora Mindfulness	RCT	Decreased depression, stress, and improved mindfulness, positive affect, social connectedness and mental health in intervention group
Shellman and Hill (2017)	132	Outdoor Education	13-day	RS, MHC-SF	Co-curricular (workshop)	NS/resilience, flourishing	NS	Quasi-experimental	Increased psychological resilience and overall mental health in intervention group
Smith et al. (2021)	288	Positive Psychology/Character Strength	16 weeks	PERMA-Profiler	Curricular (seminar)	PERMA Model/PERMA, happiness	NS	Quasi-experimental	Increased happiness and well-being in intervention group
Tolcher et al. (2024)	132	Gratitude	8-weeks	SWLS, WHO-5, OHQ, GQ-6, BRS, PANAS, DASS-21	Co-curricular (self-practice)	NS/gratitude	NS	Quasi-experimental	Increased well-being in intervention group
Wingert et al. (2022)	52	Mindfulness-Based Strengths Practice	8-week	PERMA-Profiler, Workplace PERMA-profiler	Co-curricular (workshop)	PERMA Model/PERMA	NS	RCT	Improved well-being, engagement, meaning, and health in intervention group
Yamada and Victor (2012)	60	Mindfulness	15-week	FMI, MAAS, SCS, RRQ, PSS, STAI	Curricular (workshop)	NS/mindful awareness, achievement		Quasi-experimental	Increased mindful awareness traits and reduced rumination and state anxiety in Mindfulness group

*Researchers who reported explicit funding. The Outcome Measurement acronyms can be found in Appendix A. Educational Context definitions; workshop: a course that requires skill-building and practice, seminar: a course that focuses on information sharing and discussion, interaction session: specific event for intervention to occur, self-practice: instructions given to participants to do a given task on their own, online videos: online items to watch that are not in a course structure. NS, “Not Specified.” RCT, “Randomized Control Trial.”

### 3.1 How well-being is defined and operationalized

Across the reviewed literature, well-being was operationalized using a variety of psychological constructs and psychometric instruments. Only seven studies (17.5%) explicitly referenced a published theory of well-being or a theoretical framework related to a specific construct of well-being. The majority, however, lacked a clear or in-depth definition of well-being, often relying on vague or generalized descriptions. Stress reduction emerged as a central aim in at least nine studies, typically assessed through standardized tools such as the Perceived Stress Scale (PSS) and the Depression Anxiety Stress Scales (DASS-21) (e.g., Baghurst and Kelley, 2024; Barker et al., 2016; Frazier et al., 2015; Hintz et al., 2015). Psychological well-being was commonly measured using instruments like the Scales of Psychological Well-Being (SPWB), the Mental Health Continuum–Short Form (MHC-SF), and assessments of positive affect and functioning, including the Satisfaction with Life Scale (SWLS) and the Oxford Happiness Questionnaire (OHQ) (e.g., Levin et al., 2016; Renshaw and Rock, 2018; Tolcher et al., 2024).

Several studies define well-being as “resilience and flourishing”(e.g., Seppälä et al., 2020; Shellman and Hill, 2017). Additional constructs linked to well-being included hope (Feldman and Dreher, 2011), self-efficacy (Hendrickse et al., 2024), resilience (Shellman and Hill, 2017), engagement and meaning (Flinchbaugh et al., 2012), and flourishing. Two studies (e.g., Smith et al., 2021; Wingert et al., 2022) explicitly operationalized flourishing within Seligman’s PERMA framework (2011), which emphasizes Positive Emotions, Engagement, Relationships, Meaning, and Achievement. These dimensions were typically assessed using validated instruments, including the PERMA-Profiler, the Gratitude Questionnaire (GQ-6), and the Connor-Davidson Resilience Scale, among others.

Mindfulness was frequently conceptualized as both a dimension and a predictor of well-being. Relevant studies utilized validated measures such as the Kentucky Inventory of Mindfulness Skills (KIMS), the Five Facet Mindfulness Questionnaire (FFMQ), and the Mindful Attention Awareness Scale (MAAS) (e.g., Bergen-Cico et al., 2013; Long et al., 2021; Yamada and Victor, 2012). Notably, studies focused on mindfulness were among those most likely to omit an explicit definition or theoretical framing of well-being.

To structure the scope of interventions, they can be generally classified into six overarching categories:

1. Psychoeducation and psychotherapy (e.g., Cognitive Behavioral Therapy, Acceptance and Commitment Therapy) (n = 10 studies, 25%);
2. Goal-setting and behavior change (n = 1 study, 2%);
3. Mindfulness and meditation-based interventions (n = 7 studies, 17%);
4. Positive psychology interventions (e.g., gratitude, character strengths, resilience-building) (n = 11 studies, 27%);
5. Interpersonal interventions (e.g., social connection, communication skills) (n = 2 studies, 5%); and
6. Physical or mind–body approaches (e.g., yoga, exercise, breathing techniques) (n = 4 studies, 10%).

Several additional studies employed unique methodologies that do not align neatly with the primary intervention categories (e.g., Barker et al., 2016). In some cases, studies span multiple categories or could be reasonably classified under more than one domain (e.g., Baghurst and Kelley, 2024; Conley et al., 2024).

### 3.2 What constitutes a well-being or mental health intervention

The reviewed interventions varied in theoretical orientation, format, and duration. Mindfulness-based interventions—including MBSR, mindfulness training, and app-based formats—were among the most frequently implemented, ranging in duration from 4 to 15 weeks (e.g., Bergen-Cico et al., 2013; Huberty et al., 2019; Yamada and Victor, 2012).

Acceptance and Commitment Therapy (ACT) was another prevalent approach, delivered through seminars and online formats over periods ranging from three sessions to several weeks (e.g., Browning et al., 2023; Firestone et al., 2019; Levin et al., 2017).

Stress management interventions included psychoeducational workshops, sport and fitness programs, and gratitude journaling, with durations from a single session to full-semester implementations (e.g., Baghurst and Kelley, 2024; Flinchbaugh et al., 2012; Nguyen-Feng et al., 2017).

Positive psychology approaches also featured prominently, emphasizing strengths, positive emotions, and happiness through in-person and online formats (e.g., Goodmon et al., 2016; Jeong et al., 2022; Tolcher et al., 2024). Other unique formats included interactions with therapy dogs (Barker et al., 2016), movement-based classes like Taijiquan (Caldwell et al., 2011), and positive media consumption (Prestin and Nabi, 2020).

### 3.3 How interventions are integrated into the undergraduate experience

Well-being interventions were integrated into the undergraduate experience through both curricular and co-curricular pathways. Curricular approaches included workshops and seminars embedded within course offerings or general education requirements (e.g., Baghurst and Kelley, 2024; Caldwell et al., 2010; Feldman and Dreher, 2011). Co-curricular interventions spanned a wide range of formats, from structured workshops to app-based programs and self-directed practices (e.g., Huberty et al., 2019; Renshaw and Hindman, 2017).

Several interventions were situated within broader institutional programs, such as *The Connection Project*, which focused on fostering a sense of belonging (Costello et al., 2022), and *The Student Resilience Project*, which aimed to strengthen self-efficacy (Hendrickse et al., 2024). Other initiatives—including *SKY Campus Happiness*, *Kora Mindfulness*, and *Foundations in Emotional Intelligence*—reflected campus-wide efforts to promote student well-being through institutional investment (Seppälä et al., 2020).

Of the 40 interventions reviewed, 14 (35%) were embedded within the academic curriculum, with participation linked to course credit or fulfillment of curricular requirements. Two additional interventions, while co-curricular in design, also offered course credit (e.g., Dalton and Estrada, 2023; Ko et al., 2018).

A few studies explicitly recommended integrating well-being initiatives into the curriculum (e.g., Browning et al., 2023; Conley et al., 2013; Smith et al., 2021), citing benefits such as enhanced legitimacy, greater scalability, and stronger alignment with institutional learning outcomes. In addition, four studies emphasized the advantages of web-based delivery, highlighting its flexibility, accessibility, and capacity for broad institutional implementation. The majority of the remaining interventions were co-curricular and typically facilitated through student affairs offices or campus counseling centers.

### 3.4 How outcomes are assessed and whether they align with intervention goals

A diverse range of outcome measures was used across the studies, reflecting the multifaceted nature of well-being interventions. These included:

- Psychological assessments, such as the Generalized Anxiety Disorder Scale (GAD), Perceived Stress Scale (PSS), and Flourishing Scale
- Physical health measures, including the Pittsburgh Sleep Quality Index (PSQI) and Youth Risk Behavior Surveillance System (YRBSS)
- Biometric and physiological indicators, such as heart rate variability and cortisol levels
- Researcher-developed instruments designed to assess specific goals unique to each intervention

A comprehensive list of validated instruments used across studies is provided in Supplementary material.

In most cases, outcome assessments were well aligned with the stated goals of the interventions. Stress-reduction programs commonly used tools like the Perceived Stress Scale (PSS) and Depression, Anxiety, and Stress Scale (DASS-21) to track changes in perceived stress and emotional distress (e.g., Barker et al., 2016; Hintz et al., 2015). Mindfulness-based interventions often measured skill development using instruments such as the Kentucky Inventory of Mindfulness Skills (KIMS), Five Facet Mindfulness Questionnaire (FFMQ), and Mindful Attention Awareness Scale (MAAS) (e.g., Caldwell et al., 2010; Ramler et al., 2015).

Programs aimed at enhancing overall well-being tended to employ broader assessments, including the Mental Health Continuum–Short Form (MHC-SF), Satisfaction with Life Scale (SWLS), and Oxford Happiness Questionnaire (OHQ) (e.g., Renshaw and Rock, 2018; Tolcher et al., 2024). Targeted constructs such as social connectedness, hope, and self-efficacy were measured using tools like the UCLA Loneliness Scale, Trait Hope Scale, and General Self-Efficacy Scale (e.g., Costello et al., 2022; Feldman and Dreher, 2011; Hendrickse et al., 2024).

Several studies employed multiple outcome measures to capture broader effects beyond the intervention’s primary focus. For example, a stress-reduction program might also assess student engagement or sense of meaning in life (Flinchbaugh et al., 2012).

### 3.5 What strategies support the implementation and sustainability of well-being initiatives, particularly regarding institutional frameworks and funding

Sustainability emerged as an underexplored aspect in the reviewed literature. Of the 40 studies, only 11 (27.5%) explicitly reported a funding source—primarily research grants—indicating limited evidence of ongoing institutional support. Few studies addressed long-term implementation plans, integration into existing campus infrastructure, or mechanisms for sustained funding and evaluation.

Implementation strategies and institutional frameworks were largely absent or only implicitly described. Only two studies clearly positioned their interventions within broader, university-wide initiatives—*The Connection Project* (Costello et al., 2022) and *The Student Resilience Project* (Hendrickse et al., 2024). Other programs, such as *SKY Campus Happiness* and *Foundations in Emotional Intelligence*, may suggest sustained institutional partnerships and funding commitments, though these connections were not always explicitly detailed (Seppälä et al., 2020).

One explicitly noted that their interventions were part of a general education wellness requirement (e.g., Caldwell et al., 2011), suggesting that well-being content had been formally integrated into the curriculum. Such curricular embedding not only enhances legitimacy but also facilitates more sustainable implementation.

A few interventions demonstrated strategic targeting of key student populations—such as first-year students (e.g., Melnyk et al., 2015)—as a way to support early adjustment and retention. Additionally, the adoption of scalable formats, including mobile apps, online workshops, and web-based platforms, was evident in several studies (e.g., Conley et al., 2024; Firestone et al., 2019), signaling a growing emphasis on accessibility and long-term sustainability.

## 4 Discussion

This scoping review is the first to systematically examine empirical well-being interventions developed specifically for undergraduate students in U.S. higher education between 2009 and 2024 through the lens of Positive Higher Education. As proposed in this paper, Positive Higher Education represents an emerging perspective that positions student well-being as a central institutional outcome—complementary to traditional academic goals. Aligned with the values of American liberal arts education, this perspective emphasizes holistic development, encompassing intellectual, emotional, social, and ethical domains.

The findings of this review underscore both the progress and the persistent limitations in the landscape of well-being interventions in U.S. undergraduate education. A key strength emerging from the 40 studies is the remarkable diversity of intervention types and delivery formats, reflecting a growing recognition of well-being as a multifaceted construct. Interventions spanned mindfulness, cognitive-behavioral therapies, positive psychology exercises, physical activity, and social connection strategies—offering a broad array of tools that institutions can adapt to varied campus contexts and student needs. These findings align with recent systematic reviews that affirm the efficacy of a variety of interventions (Barnett et al., 2021; Conley et al., 2015, 2016, 2017; Fenton et al., 2018; Winzer et al., 2017, 2018). Among the studies covered in the current review, many demonstrated a thoughtful alignment between their intended outcomes and the measures employed, suggesting a maturing field that is attentive to methodological rigor. The use of validated psychometric instruments and, in some cases, physiological and behavioral markers, provided robust evidence of the effectiveness of these interventions, particularly in reducing stress and enhancing psychological well-being. Moreover, the incorporation of digital platforms, mobile and even robotic applications, reflects a promising trend toward scalable, accessible solutions that meet students where they are.

At the same time, several notable gaps limit the broader impact and generalizability of this body of research. Chief among these is the conceptual ambiguity surrounding the definition of well-being, echoing the findings of other reviews (du Toit et al., 2022; Dodd et al., 2021). More than half of the studies lacked a clear or theoretically grounded definition, often defaulting to stress reduction or symptom relief as proxies. This absence of a unifying conceptual framework hinders cross-study comparisons and slows the development of a coherent, evidence-based approach to Positive Higher Education. While a handful of studies employed a well-being model such as PERMA (Seligman, 2011), or a framework related to a specific construct like hope theory (Snyder et al., 2002), these were exceptions rather than the norm.

Furthermore, although some interventions were embedded in academic courses or linked to curricular requirements—enhancing their legitimacy and reach—the majority remained co-curricular and peripheral to the academic core. Curricular interventions simultaneously offer broader incentives for students to enroll while increasing institutional sustainability. Well-being content can be meaningfully integrated into teaching and learning, but doing so requires cultural and structural shifts that may be difficult to enact within decentralized, discipline-driven university environments. If the goal of the Positive Higher Education paradigm is for every student to demonstrate knowledge, skills, attitudes, or aptitudes related to well-being—and for these to be recognized as essential outcomes of higher education—then determining where and how this content is most appropriately embedded becomes an important and pressing discussion.

A final and critical limitation lies in the limited attention to institutionalization and sustainability. Organizational barriers at undergraduate universities can make it difficult to implement these new well-being initiatives. These may include faculty burnout, a crowded curriculum with no space for new classes, and/or resource constraints that preclude the adoption of new costly interventions. Indeed, few studies described long-term funding mechanisms, strategic alignment with institutional priorities, or integration into broader campus frameworks. Without these supports, even effective interventions risk being short-lived or siloed. Although some promising examples—such as *The Connection Project* (Costello et al., 2022) and the *Student Resilience Project* (Hendrickse et al., 2024)—demonstrate the potential of institutionally backed initiatives, these remain relatively rare. Future research should not only continue to explore the efficacy of interventions but also attend more fully to questions of scalability, policy integration, and systemic change. A more cohesive research agenda grounded in positive education principles, and sensitive to the organizational realities of higher education, will be essential for realizing the full potential of well-being as a central aim of undergraduate education.

### 4.1 Practical implications

Drawing from the findings of this scoping review, several implications can guide research, practice, and policy through the lens of Positive Higher Education. First, institutions should strive for holistic educational outcomes that integrate academic achievement with personal development, character formation, and student flourishing. This broader vision of higher education supports not only cognitive growth but also emotional, social, and ethical development—and, in the context of American higher education, aligns closely with the values and aims of the liberal arts college model.

Second, the adoption of a strengths-based approach is critical. Interventions should be designed to help students identify, develop, and apply their individual strengths, fostering greater engagement, intrinsic motivation, and psychological resilience. In addition, well-being literacy should be considered a core educational competency. All students should graduate with a foundational understanding of well-being, including the skills, attitudes, and dispositions necessary to sustain their own and others’ flourishing in personal, academic, and professional domains.

Lastly, a systems-level perspective is necessary for integrated implementation. Well-being should be embedded across curriculum design, pedagogy, co-curricular activities, institutional structures, and policy. Such integration enables institutions to transition from fragmented, reactive programming to cohesive, proactive strategies that promote well-being as a shared and sustained institutional responsibility.

These principles offer a conceptual foundation for future scholarship and a practical framework for institutional planning and assessment, supporting a comprehensive and inclusive approach to student well-being in higher education.

### 4.2 Limitations and future directions

While this review provides a valuable synthesis of well-being interventions in U.S. undergraduate education, several limitations should be acknowledged. The exclusive focus on empirical studies within the United States limits the generalizability of findings to international and cross-cultural contexts. To contextualize these findings, it is important to note that the U.S. higher education system’s unique student culture—shaped in part by its emphasis on general education and liberal arts curricula—and its decentralized institutional structures differ significantly from those in other regions, such as Asia and Europe, thereby influencing how well-being interventions are conceptualized, designed, and implemented. Additionally, the exclusion of non-empirical sources—such as institutional policy documents, planning frameworks, and gray literature—constrains our understanding of system-level strategies for embedding well-being in higher education.

As a scoping review, this study is limited in its scope by design, focusing on the conceptualization, operationalization, and measurement of well-being rather than evaluating the methodological quality or efficacy of individual interventions. The findings from included studies were mapped and summarized but not critically appraised, analyzed for effectiveness, or synthesized through meta-analytic techniques. While enhancing the efficacy of well-being interventions in higher education is an important area for future inquiry, such analysis falls outside the aims and methodological parameters of this review. Further research employing rigorous evaluative frameworks is warranted to assess intervention effectiveness and inform evidence-based practice.

Another limitation is the predominance of short-term, small-scale studies. Study design has important implications for determining causality. Specifically, more confidence can be placed in the effectiveness of an intervention if there is a control group and randomization. It is less clear if the intervention itself led to improvements in student well-being when these study design factors are missing. In addition, few interventions were evaluated over extended timeframes or implemented at the institutional level, which limits insight into the sustainability, scalability, and long-term outcomes of these efforts. To address this gap, a follow-up qualitative study is currently underway, examining case studies of institutions that have formally adopted well-being as a strategic objective. This research explores how well-being is conceptualized, measured, and operationalized across governance, curriculum, student services, and faculty development.

A key recommendation arising from this review is the need for a *Positive Higher Education Implementation Toolkit*—a resource to guide institutions in embedding well-being into the core of university operations. This toolkit should include: (1) a clearly defined yet adaptable framework for Positive Higher Education; (2) design and evaluation guidelines for interventions; (3) a curated set of validated measurement instruments; (4) examples of curricular, co-curricular, and institutional best practices; and (5) strategies for long-term sustainability, stakeholder engagement, and policy alignment. The creation of such a toolkit could be undertaken by a collaborative group of scholars and practitioners, ideally supported by the International Positive Education Network in partnership with other leading organizations. This recommendation is informed in part by the Healthy Minds Network’s current initiative to develop a “repository of what works” for student mental health in higher education.^1^ A Positive Higher Education Toolkit would complement such efforts by offering a broader, integrative approach, supporting institutions in conceptualizing and operationalizing well-being as a core dimension of their educational mission.

In parallel, there is an urgent need for national organizations and initiatives—such as the Okanagan Charter (2015) and Bringing Theory to Practice—to reexamine their foundational policy frameworks in light of shifting student demographics, increasing mental health concerns, and an evolving global geopolitical landscape. The findings of this review call for a deeper collective reflection and system-wide dialogue, among students, staff, faculty, administrators, and policy makers, around the role of well-being in higher education. Beyond isolated programs or interventions, institutions must consider what it truly means to position well-being as a core educational outcome—on par with disciplinary knowledge, critical thinking, and civic responsibility.

## 5 Conclusion

This scoping review contributes to the growing literature on student well-being by offering a targeted synthesis of empirical well-being interventions in U.S. undergraduate education, interpreted through the lens of Positive Higher Education. While the field has expanded significantly over the past decade, the findings reveal persistent challenges—particularly around conceptual coherence, consistent measurement, and systemic implementation.

Looking forward, there is a clear need for coordinated, theory-informed approaches that embed well-being as a central educational priority. To support this evolution, we advocate for the creation of a Positive Higher Education Implementation Toolkit to guide institutions in designing, integrating, and evaluating well-being initiatives. Such a resource would help transform isolated efforts into strategic, institution-wide practices aligned with contemporary student needs and the educational mission of higher education.

As colleges and universities navigate ongoing challenges and shifting expectations, this is a pivotal moment to elevate well-being from a peripheral concern to a central institutional commitment—grounded in evidence, informed by theory, and guided by a vision of higher education that places human flourishing at its core.

## Footnotes

^1^ https://healthymindsnetwork.org/best-practices-repository/
