# Supplementary material for: Well-being interventions in U.S. colleges: a scoping review from a positive higher education perspective
Source: Front Psychol. 2025 Aug 29;16:1601955. doi: 10.3389/fpsyg.2025.1601955 (PMC12426774; doi:10.3389/fpsyg.2025.1601955)
Supplement: Supplementary file 1 [file Table_1.docx]

**Appendix A**

**Table A1**
*Acronyms, Measure Full Names, and Subject Areas*

| **Acronym** | **Measure Full Name** | **Subject** |
| --- | --- | --- |
| 5F-WEL | Five Factor Wellness Evaluation of Lifestyle | Wellness |
| AAQ | Acceptance and Action Questionnaire | Experiential Avoidance |
| AAQ-II | Acceptance & Action Questionnaire-II | Psychological Inflexibility |
| ADHS | Adult Dispositional Hope Scale | Hope |
| AFQ-Y | Avoidance and Fusion Questionnaire for Youth | Psychological Inflexibility and Flexibility for Youth |
| AHI | Authentic Happiness Inventory | Happiness |
| AHQ | Approaches to Happiness Questionnaire | Happiness |
| AHS | The Adult Hope Scale | Hope |
| ATQ-P | Automatic Thought Questionnaire—Positive | Automatic Thought |
| ATQ-SF | Adult Temperament Questionnaire - Short Form | Temperament |
| AUDIT | Alcohol Use Disorders Identification Test | Alcohol |
| BAIT | Beck Anxiety Inventory-Trait | Anxiety |
| BDI | Beck Depression Inventory | Depression |
| BFNE | Brief Fear of Negative Evaluation Scale | Anxiety about Social Evaluation |
| BMIS | Brief Mood Introspection Scale | Mood-related |
| BRS | Brief Resilience Scale | Recovery from Stress |
| CAS | College Adjustment Scales | Adjustment Difficulties among College Students |
| CAMS-R | Cognitive & Affective Mindfulness Scale -Revised | Trait Mindfulness |
| CCAPS-34 | The Counseling Center Assessment of Psychological Symptoms | Broad Range of Mental Health Problems |
| CD-RS | Connor-Davidson Resilience Scale | Resilience |
| CEI-II | Curiosity and Exploration Inventory | Intellectual Curiosity and Exploration |
| CES-D | The Center for Epidemiological Studies-Depression | Depression |
| CFQ | Cognitive Fusion Questionnaire | Compassion |
| CLS | The Compassionate Love Scale | Compassion |
| COPE | COPE Inventory | Coping Behaviors |
| CS | The Compassion Scale | Compassion |
| DAS | Dysfunctional Attitudes Scale | Attitudes |
| DASS-21 | Depression Anxiety and Stress Scale-21-item | Depression, Anxiety, Stress |
| DES | Difficulties in Emotion Regulation Scale | Emotion Regulation |
| DES | Dispositional Envy Survey | Envy |
| EQ | Experiences Questionnaire | Decentering and Rumination |
| ERQ | Emotion Regulation Questionnaire | Emotion Regulation |
| FDMS | Four Dimensional Mood Scale | Mood |
| FFMQ | Five Facet Mindfulness Questionnaire | Mindfulness |
| FFMQ-15 | Five Facet Mindfulness Questionnaire | Mindfulness |
| FMI | Freiburg Mindfulness Inventory | Mindfulness |
| GAD | Generalized Anxiety Disorder Scale | Anxiety Screening |
| GHQ | General Happiness Questionnaire | Happiness |
| GLES | General Life Events Schedule | Stressful Life Events |
| GSHS | Goal-Specific Hope Scale | Hope/Goal Setting |
| GQ-6 | Gratitude Questionnaire | Dispositional Gratitude |
| ICSRLE | Inventory of College Students’ Recent Life Experiences | Experiences |
| IRI | Interpersonal Reactivity Index | Empathy |
| KIMS | Kentucky Inventory of Mindfulness Scale | Mindfulness |
| LOT-R | Life Orientation Test–Revised | Value |
| MAAS | Mindful Attention Awareness Scale | Mindfulness |
| MASQ-D30 | The Mood and Anxiety Symptom Questionnaire | Mood |
| MBI | Maslach Burnout Inventory | Burnout |
| MHC-SF | Mental Health Continuum-Short Form | Positive Mental Health Dimensions |
| Mini-IPIP | International Personality Item Pool Scale | Personality Traits |
| MLQ | Meaning of Life Questionnaire | Meaning |
| NCS | National Comorbidity Survey | Suicidal Ideation and History |
| OHQ | Oxford Happiness Questionnaire | Happiness |
| PANAS | Positive & Negative Affect Schedule | Positive and Negative Affect |
| PBS | The Personal Burnout Scale | Burnout |
| PBS | Personal Beliefs Scale | Beliefs about the Ability to Manage |
| PCI | Perfectionistic Cognitions Inventory | Perfectionism |
| PCOSES | Present Control Subscales | Present Control |
| PERMA-Profiler | PERMA Profiler | Wellbeing PERMA Dimensions |
| PHLM | Philadelphia Mindfulness Scale | Mindfulness |
| PHQ | Primary Health Questionnaire | Depression Screening |
| PHQ-9 | The Patient Health Questionnaire-9 | Depression |
| PILT | Purpose in Life Test | Life Meaning/Purpose |
| PROMIS | Patient-Reported Outcomes Measurement Information System | Physical, Sleep |
| PSQI | Pittsburgh Sleep Quality Index | Sleep |
| PSS | Perceived Stress Scale | Stress |
| PSS-4 | The Perceived Stress Scale–4 | Stress |
| PSS-10 | The Perceived Stress Scale–10 | Stress |
| PSS-14 | The Perceived Stress Scale–14 | Stress |
| PSSM | Psychological Sense of School Membership Scale | Belongingness |
| PSWQ | Penn State Worry Questionnaire | Worry/Anxiety |
| PVQ | Personal Values Questionnaire | Values Across Nine Life Domains |
| PWB | Ryff's Psychological Well-being Scale | Psychological Well-being |
| Q-LES-Q-SF | Quality of Life Enjoyment & Satisfaction Questionnaire Short Form | Satisfaction and Enjoyment in Daily Life |
| QOLI | Quality of Life Inventory | Satisfaction of Life |
| RAND-SF36 | The RAND Corporation’s 36-Item Short Form Health Survey | Quality of Life |
| RS | Resilience Scale | Resilience |
| RSE | Rosenberg Self-esteem Scale | Self-esteem |
| RPWS | Ryff's Psychological Wellbeing Scale | Wellbeing |
| RRQ | The Rumination-Reflection Questionnaire | Depressive Rumination |
| RTCR | Readiness to Change Ruler | Willingness to Change |
| SACQ | Student Adjustment to College Questionnaire | Adjustment |
| SAS | Self-actualization Assessment | Self-Actualization |
| SBI | Savoring Beliefs Inventory | Savoring |
| SCC-R | The Social Connectedness Scale | Connectedness |
| SCS | The Self-Compassion Scale | Self-compassion |
| SCS-SF | The Self-Compassion Scale-Short Form | Self-compassion |
| SES-GSE | The Self-efficacy Scale: General Self-efficacy Subscale | Self-efficacy |
| SHS | The Subjective Happiness Scale | Happiness |
| SISA | The Short Index of Self-Actualization and Purpose in Life | Self-actualization |
| SISE | The Single Item Self-Esteem Scale | Self-esteem |
| SRSE | Self-Regulatory Self-Efficacy Scale | Self-efficacy |
| STAI | The State-Trait Anxiety Inventory | Anxiety |
| STAI-T | Spielberger State-Trait Anxiety Inventory-Trait Form Y-2 | Trait Anxiety |
| SWLS | The Satisfaction with Life 5-item Scale | Life Satisfaction |
| TAS | The Test Anxiety Survey | Anxiety |
| THS | Trait Hope Scale-12 | Hope |
| TLEQ | Traumatic Life Events Questionnaire | Trauma |
| UCLA Loneliness | The UCLA Loneliness Scale | Loneliness |
| UCLA Loneliness-R | UCLA Loneliness Scale–Revised | Loneliness |
| UFM | Unified Flexibility and Mindfulness Scale | Psychological Flexibility |
| URICA-Short Form | University of Rhode Island Change Assessment-Short Form | Readiness to Change |
| VIA-IS | VIA Inventory of Strengths | Character Strengths |
| VIQ | Vocational Identity Questionnaire | Vocational Calling |
| VLQ | Valued Living Questionnaire | Values |
| VQ | Valuing Questionnaire | Values |
| WAI-SR | Working Alliance Inventory-Short Revised | Rapport |
| WHO-5 | World Health Organization-Five Well-Being Index | Well-being |
| Workplace PERMA-profiler | Workplace PERMA-profiler | Well-being |
| YAAPST | Young Adult Alcohol Problems Screening Test | Drinking Behavior |
| YRBS | Youth Risk Behavior Surveillance | Physical, Drinking, Eating |
| ZKPQ-Form III Inf | Zuckerman-Kuhlman Personality Questionnaire-Form III | Attention |
